# Supplementary material for: Surface Microbiome Profiling of Dental Elevators Using Third‐Generation Sequencing: Implications for Infection Control in Dental Practice
Source: Microbiologyopen. 2025 Dec 8;14(6):e70178. doi: 10.1002/mbo3.70178 (PMC12685768; doi:10.1002/mbo3.70178)
Supplement: Supplementary file 1 — customer_backup. [file MBO3-14-e70178-s001.zip › customer_backup/customer_backup/taxa_summary/krona/groups/treat/M01.Krona.html]

Javascript must be enabled to view this page.

members
magnitude
magnitudeUnassigned

M01.krona

55481

55477

213

0

0

0

0

0

0

0

0

0

0

0

0

0

0

0

213

213

213

213

54

2

27

0

9

49

28

6

38

0

0

0

0

0

0

0

0

0

0

0

0

0

0

1918

1802

3

3

3

3

12

12

2

2

0

0

0

10

10

0

0

0

0

1787

0

0

0

0

0

0

0

0

0

0

0

0

0

0

0

0

2

1

1

0

0

0

0

1

1

0

1785

1784

1784

1

1

116

0

0

0

0

0

0

0

116

5

5

5

22

22

22

5

5

5

16

16

16

68

68

68

305

267

267

0

0

0

267

166

164

2

81

26

51

0

4

20

20

0

0

0

0

0

0

0

0

0

0

0

0

0

0

0

0

0

0

0

0

0

0

0

0

0

0

0

0

0

0

0

0

0

0

0

0

0

0

0

0

0

0

0

0

0

0

0

0

0

0

0

0

0

0

0

0

0

0

0

0

0

0

0

0

0

0

0

0

0

0

0

0

0

0

0

0

0

0

0

0

0

0

0

0

38

26

26

0

0

26

26

0

0

0

0

0

0

0

0

0

0

0

0

0

12

12

12

12

0

0

0

0

0

0

0

0

0

0

0

0

0

0

0

3

3

3

3

3

3

0

0

0

0

0

0

0

0

3

3

3

0

0

0

0

0

0

3

3

3

0

0

0

0

0

0

423

4

4

4

0

0

2

2

1

0

1

1

1

0

3

3

3

3

3

416

416

416

41

28

13

366

0

1

130

0

235

1

1

8

8

0

1025

252

252

0

0

0

252

252

252

0

0

0

0

0

0

0

0

0

0

0

0

0

0

0

0

773

536

448

426

113

237

76

22

15

6

1

0

88

88

87

1

12

0

0

0

12

12

12

0

0

196

10

2

2

0

0

8

0

8

0

0

0

0

0

0

0

2

0

0

0

2

0

2

0

0

184

184

62

122

29

29

0

0

0

0

3

3

26

7

19

0

0

0

0

0

0

0

18038

6532

0

0

0

0

104

6

6

6

97

1

1

0

0

0

0

0

0

96

1

95

0

1

1

1

490

490

240

0

240

246

246

4

4

73

0

0

0

73

22

22

0

0

2

2

49

49

0

0

0

135

135

13

5

7

1

122

4

5

1

112

0

0

0

0

0

0

0

685

685

0

0

18

1

3

1

1

12

0

0

2

2

0

0

1

1

0

0

0

0

93

93

29

2

4

9

5

1

4

4

0

0

106

12

49

26

19

4

0

4

360

0

0

0

0

5

0

5

0

0

13

0

0

22

57

0

0

6

51

21

1

96

0

3

1

55

23

1

0

0

0

72

46

2

0

24

6

6

6

6

0

0

0

0

0

4459

158

0

0

68

1

67

2

2

0

0

0

6

3

3

82

28

22

32

0

0

0

69

12

4

8

38

7

16

0

1

4

0

1

9

19

19

15

15

15

1

1

1

13

13

13

24

24

24

0

0

0

1138

0

0

0

0

0

0

0

368

337

31

2

0

2

79

79

0

0

0

0

0

10

10

535

2

375

31

87

33

0

7

0

0

143

3

3

0

0

36

27

32

2

10

15

3

12

1

1

134

10

10

1

1

53

53

70

70

0

0

0

0

0

26

26

26

0

0

0

12

12

12

237

39

1

5

33

0

0

0

3

3

195

41

137

2

15

194

142

142

0

52

52

0

1526

35

12

23

158

158

448

448

13

11

2

4

4

4

4

9

9

0

0

855

58

797

138

138

1

137

774

49

49

0

0

0

0

32

0

2

30

0

0

576

47

169

7

0

124

2

2

0

0

33

190

2

0

0

3

3

20

12

3

4

1

3

3

0

0

40

40

51

51

0

0

0

0

0

0

94

25

9

9

16

16

69

46

3

11

7

25

7

0

7

10

1

3

5

1

2

2

4

1

3

0

11

11

11

11

1

1

1

0

0

1

0

0

0

0

0

0

10

10

10

10

0

0

0

0

0

0

0

0

20

20

20

17

3

2

2

2

2

0

0

0

0

0

0

0

0

0

0

0

0

0

0

255

255

0

0

17

17

0

90

90

19

2

7

8

1

0

0

0

1

0

0

38

3

27

8

4

4

70

10

52

8

0

0

17

17

0

0

0

0

37

9

9

9

0

0

0

6

6

6

8

8

8

2

2

2

2

2

2

2

2

2

1

1

1

7

7

7

150

150

0

0

0

0

0

0

0

0

0

0

0

0

0

0

0

0

0

0

0

0

0

0

0

0

0

44

31

13

0

0

0

0

4

0

0

3

0

1

29

12

9

8

0

0

2

2

71

20

51

0

11506

0

0

0

0

0

0

0

0

0

0

0

0

47

43

43

43

4

4

4

8591

20

1

1

19

19

1

0

0

0

0

1

1

11

11

0

11

0

0

0

0

0

0

0

0

14

14

14

64

64

64

102

99

99

3

3

2402

6

6

7

7

2161

2161

188

13

0

90

85

40

1

0

1

1

0

14

1

0

2

0

4

0

4

2

0

10

102

4

4

28

28

42

5

15

22

20

20

5

5

3

0

3

3

3

3

36

1

1

3

3

3

3

1

0

1

0

28

28

0

0

0

0

77

1

1

0

0

0

0

0

76

0

76

0

135

135

135

5485

0

0

4

3

0

1

20

20

139

139

0

0

134

2

42

90

38

33

5

11

11

384

33

351

0

0

17

1

16

12

0

1

11

27

27

17

0

17

4677

4677

5

5

139

84

84

55

44

11

246

219

140

124

16

68

68

11

11

27

27

26

1

0

0

0

0

36

36

36

36

0

0

0

0

0

0

0

0

0

0

0

0

0

0

0

0

1

1

1

1

0

0

0

0

0

0

0

0

0

0

0

113

0

0

0

15

1

1

0

0

0

0

0

1

1

13

0

0

1

12

0

0

0

0

0

0

0

0

25

25

0

25

0

0

0

0

0

0

0

0

0

73

73

2

0

67

4

0

0

0

0

0

0

0

0

0

0

0

0

0

0

0

0

0

0

0

0

0

0

0

0

0

0

0

58

58

0

0

0

0

58

58

0

0

0

0

0

0

0

0

0

0

0

0

0

0

0

0

0

0

0

0

0

0

0

2064

3

2

2

1

1

1

1

1

5

0

0

0

5

5

0

0

0

1939

1826

1826

12

12

81

81

20

20

3

3

2

1

101

101

101

5

5

5

6

6

6

1

1

1

0

0

0

0

0

0

0

0

0

0

0

0

0

0

0

0

0

0

0

0

0

0

0

0

0

5

5

5

2

0

0

3

0

0

0

0

0

0

0

0

0

0

0

0

0

0

0

194

37

2

2

4

4

0

3

3

11

11

17

3

14

157

17

17

4

4

0

0

15

0

15

71

10

1

60

0

50

4

3

0

0

0

3

0

0

0

6

32

1

1

0

0

0

0

0

0

0

0

0

0

0

0

0

0

0

0

0

0

0

0

0

0

150

150

139

11

93

35

0

0

0

0

0

0

0

0

11

11

0

0

0

0

0

0

0

0

0

0

0

0

0

0

0

0

0

0

0

0

0

0

0

0

0

0

0

0

0

1

1

1

1

0

533

125

125

0

0

0

125

6

6

4

4

1

1

19

19

53

0

53

2

2

0

0

40

38

0

2

408

0

0

0

0

0

0

0

1

0

0

0

0

0

0

1

1

1

173

65

9

4

5

9

9

0

8

1

7

39

0

9

30

0

0

0

0

0

0

0

90

1

0

1

89

0

64

25

0

18

6

6

12

12

0

0

0

0

193

193

193

5

19

91

78

0

0

0

0

0

0

0

41

41

41

41

0

0

0

0

0

0

0

0

0

0

4

4

4

4

4

0

1

2

1

0

0

0

0

0

0

4093

0

0

0

0

0

4087

506

11

11

11

495

2

0

2

31

0

10

21

63

33

1

24

5

115

5

106

4

47

47

1

1

44

1

7

5

11

1

14

0

5

0

72

9

13

32

0

18

0

0

3

3

0

0

0

0

39

39

3

3

71

2

69

0

4

4

0

0

0

0

0

0

0

0

67

0

0

0

46

0

0

0

0

0

0

0

0

21

0

0

21

0

0

0

0

0

0

13

1

12

0

0

0

0

0

0

0

0

12

12

0

0

0

0

0

0

0

0

0

0

0

0

0

0

8

8

0

8

0

0

0

0

0

13

0

0

0

0

13

13

0

0

3350

0

0

0

0

0

11

11

11

0

0

0

1114

8

8

44

44

356

356

706

706

271

0

0

0

5

5

59

0

0

59

0

0

0

0

207

3

70

83

19

3

2

0

27

0

0

0

0

0

253

6

6

0

0

247

17

34

196

0

0

0

0

0

0

0

0

989

989

0

1

6

53

788

0

2

0

1

0

22

0

4

0

0

0

112

518

95

95

0

0

0

423

13

127

90

97

2

94

193

0

0

0

50

0

50

86

0

0

86

0

0

55

55

1

1

0

0

1

0

1

0

0

0

0

0

0

0

0

0

0

0

0

0

0

0

0

0

0

0

0

0

0

0

0

0

0

0

0

0

0

0

0

0

0

0

0

0

0

1

1

1

0

0

0

0

0

0

0

0

0

0

0

6

0

0

0

6

4

3

0

1

2

2

0

0

0

0

0

0

0

0

0

0

0

0

0

0

0

158

24

0

0

1

1

0

0

23

0

5

18

16

0

0

16

16

0

0

0

0

0

0

1

0

0

0

0

0

0

0

0

0

1

1

0

42

42

42

53

14

14

27

27

12

0

10

2

22

0

0

6

0

0

1

2

3

0

11

11

0

0

0

1

0

1

4

0

0

0

0

0

0

1

0

0

1

0

1

1

0

0

0

0

0

0

0

0

0

0

0

0

1

1

0

0

0

1

1

1

5

0

0

0

0

0

0

0

0

0

0

5

5

5

5

232

147

5

5

0

0

0

0

0

5

0

5

0

0

0

0

0

0

0

0

0

0

0

0

0

0

0

0

0

142

141

141

141

1

1

1

0

0

0

0

85

85

85

50

39

11

24

24

11

7

4

0

0

0

0

25

25

25

25

25

25

0

0

10438

430

430

1

1

1

0

0

0

0

0

0

0

0

0

0

0

429

0

0

50

0

48

2

0

0

0

69

69

9

9

0

0

0

0

0

0

174

13

160

1

127

3

123

1

0

0

156

156

156

156

156

1238

215

62

0

0

0

0

0

0

35

35

0

0

25

25

2

2

153

153

0

153

194

33

33

33

10

10

10

9

9

9

21

21

21

87

87

87

34

34

34

37

0

0

0

0

0

0

0

0

0

0

0

0

37

37

37

0

0

0

0

0

0

0

792

2

2

1

1

0

0

0

0

0

0

0

145

145

1

11

133

494

34

21

13

445

25

420

15

2

13

151

0

0

14

14

137

137

6048

4332

4

4

4

1642

1303

5

586

0

204

41

389

78

0

0

24

2

22

255

30

18

207

13

10

3

0

47

47

2686

68

68

0

0

2593

2593

7

7

18

18

1716

1113

1113

183

885

45

603

16

16

24

24

563

563

2324

0

0

0

0

1

1

1

1

0

0

0

0

0

0

0

0

0

0

0

0

151

151

5

5

3

3

15

6

9

10

10

11

11

0

0

43

9

2

3

29

29

0

25

4

18

18

0

0

1

1

16

16

3

3

0

0

0

0

0

0

3

3

159

1

1

0

0

0

1

0

0

135

135

34

34

19

48

23

20

3

17

1

1

2

2

0

0

109

109

0

0

0

109

0

70

1

0

0

19

0

19

0

0

152

18

0

0

16

16

2

2

13

0

0

13

13

13

13

0

13

20

20

20

22

22

3

19

66

6

6

1

1

59

59

62

5

4

0

4

1

1

57

1

1

56

56

0

0

0

0

0

0

0

0

0

0

0

0

0

0

0

0

0

0

0

0

0

19

19

19

19

0

0

0

28

28

1

1

27

2

0

24

0

1

0

0

541

293

45

45

0

0

0

0

0

0

0

0

0

0

0

14

2

12

1

0

1

0

233

233

248

29

0

23

6

0

0

166

0

0

29

15

2

2

0

8

0

0

0

0

43

0

10

11

1

0

9

0

11

1

24

39

39

0

0

0

14

14

416

416

0

0

0

0

27

13

7

4

3

3

3

363

3

39

21

54

166

80

0

0

0

0

0

12

12

10

10

1

1

683

172

1

1

0

0

9

0

9

1

1

3

0

3

89

9

55

6

19

0

4

4

15

15

50

50

202

9

9

6

6

1

1

0

0

0

186

13

173

0

0

0

0

0

0

0

28

27

26

0

1

1

1

265

4

4

1

1

2

2

47

1

46

32

32

0

0

0

0

5

5

19

19

0

0

0

0

4

4

137

4

23

110

4

4

9

9

1

1

0

0

1

1

1

15

0

0

15

15

242

242

242

242

5

134

99

4

5

4

4

4

0

0

4

0

0

0

0

0

0

0

0

0

0

1

0

0

3

0

0

0

0

0

0

0

0

0

0

0

0

0

0

0

0

0

0

1

1

1

1

1

440

440

440

440

440

440

87

87

87

86

13

13

0

0

0

0

73

1

72

1

1

1

0

0

0

0

0

0

0

0

90

90

90

89

89

0

63

26

0

0

1

1

0

1

0

0

0

0

0

0

0

0

0

0

5

0

0

0

0

0

0

0

0

0

0

0

0

0

0

0

5

5

1

1

1

0

0

0

4

4

4

38

38

38

38

1

1

0

37

37

1229

687

57

57

57

57

630

170

130

96

34

2

2

28

28

10

10

460

10

10

109

109

12

12

329

329

343

302

302

302

56

199

40

7

8

0

0

0

8

8

8

33

33

9

9

8

1

1

6

8

0

8

4

2

0

2

4

3

1

0

17

3

3

3

3

14

14

14

14

0

0

0

0

98

98

98

0

0

98

2

12

7

0

3

1

0

73

0

0

0

0

0

84

84

13

13

13

5

5

5

66

66

66

0

0

0

0

17

0

0

0

0

0

0

0

0

0

0

0

0

0

0

0

0

0

0

0

0

0

0

0

0

0

0

0

0

0

0

0

0

0

0

0

0

0

0

0

0

0

0

0

0

0

0

0

0

0

0

0

0

0

0

0

0

0

0

0

0

0

0

0

0

0

0

0

0

17

17

1

1

1

2

2

2

0

0

0

0

0

0

0

0

2

2

2

1

0

0

1

0

1

0

0

0

0

0

0

0

0

3

3

3

8

8

8

0

0

0

0

0

0

0

0

0

0

0

0

0

0

0

1

1

1

1

0

0

1

0

1

0

0

0

0

0

126

42

2

2

1

1

1

0

1

0

0

0

0

40

32

32

32

3

3

3

0

0

0

1

1

1

3

3

3

1

1

1

58

58

58

58

58

26

26

26

0

0

26

26

0

0

0

0

0

0

1

1

1

1

1

0

1

0

0

0

0

0

0

0

0

0

0

0

0

0

0

0

0

0

0

0

0

0

15589

7598

29

29

6

6

0

2

2

21

3

1

0

1

0

16

19

19

0

0

19

19

133

1

1

1

6

6

6

126

126

126

0

0

40

40

40

7

33

0

5440

5435

2

2

0

0

0

0

0

0

0

117

16

101

0

0

0

154

145

0

9

0

0

4

4

0

58

58

0

0

0

0

3

3

0

0

19

15

4

0

39

2

0

37

0

0

291

0

0

0

1

11

4

0

0

0

166

5

8

39

0

8

0

29

13

6

1

0

0

0

0

14

0

14

0

0

64

0

48

16

1604

666

1

0

933

0

4

420

0

0

0

0

420

0

0

0

0

8

8

534

71

0

1

458

4

141

141

0

0

0

0

0

0

0

0

0

0

0

0

0

0

72

72

46

46

266

0

3

121

142

0

0

15

15

146

146

1

1

31

31

43

31

12

0

0

0

109

36

73

3

3

0

1231

28

809

5

32

8

99

114

15

4

64

35

18

0

0

0

5

5

5

0

0

0

0

342

224

0

0

62

37

25

0

0

0

0

0

0

1

1

0

0

0

143

0

69

0

0

74

18

18

4

4

4

112

3

3

0

0

1

1

0

0

0

0

15

15

93

71

22

2

0

0

1

1

0

0

0

1

1

0

0

0

0

33

33

0

0

0

0

31

3

0

1

0

13

14

0

2

2

16

4

4

4

0

0

0

0

0

0

1

1

1

11

11

11

0

0

0

0

0

3

3

3

3

1393

684

27

0

27

0

0

268

268

1

1

0

1

1

0

0

5

0

5

0

0

18

18

0

0

45

33

5

3

4

61

2

0

0

56

3

39

1

1

0

13

0

0

0

24

0

8

8

0

33

33

25

0

25

153

0

0

0

153

179

179

179

0

0

0

36

7

0

7

16

15

0

1

0

13

13

3

3

3

1

1

1

8

1

1

7

7

482

107

12

5

0

3

87

0

40

40

0

0

1

1

0

0

1

0

1

120

42

29

14

35

198

198

0

0

0

15

9

6

0

0

0

150

20

20

20

0

0

0

130

130

130

0

0

0

0

0

0

7923

0

0

0

0

2684

610

14

14

18

18

7

7

99

80

2

6

2

9

262

9

253

11

11

113

113

86

86

0

0

2074

0

0

0

0

0

1

1

2073

28

0

0

0

45

26

0

0

2

1703

110

33

48

0

0

75

3

0

0

0

0

1

1

0

0

0

1

1

0

0

1931

407

0

0

0

0

119

119

208

131

0

0

0

74

3

0

0

0

0

80

52

28

0

0

1524

10

10

245

245

138

27

111

28

1

27

617

598

19

6

6

0

0

241

241

128

7

121

1

1

37

37

0

0

50

50

23

23

0

0

0

0

6

6

6

6

757

756

1

1

0

0

755

4

751

1

1

1

0

0

0

0

0

0

0

365

365

0

0

0

0

298

8

8

7

0

1

7

0

3

10

171

0

0

0

0

0

0

0

0

13

9

20

0

0

1

40

0

0

0

14

14

53

44

9

0

30

13

13

13

0

0

0

10

10

10

7

7

7

779

779

779

775

4

1369

6

6

2

4

71

2

2

0

0

68

68

1

1

0

0

145

139

0

1

2

0

101

0

15

16

0

0

0

4

0

6

6

0

30

30

1

29

1116

0

0

1

1

10

8

0

2

5

0

3

2

312

1

197

0

0

0

114

7

7

492

414

0

0

0

0

78

21

21

245

0

0

245

11

6

5

4

4

8

6

2

0

0

0

1

0

0

0

0

1

1

1

1

1

0

0

0

1

0

0

0

0

0

0

0

0

0

0

0

0

0

0

0

0

0

0

0

0

6

6

6

6

6

62

0

0

0

0

0

0

62

9

9

9

20

0

0

0

0

0

0

20

20

0

0

0

0

0

0

0

0

0

0

0

12

1

1

0

0

0

11

10

1

0

0

0

0

0

21

21

21

0

0

0

0

0

151

14

13

13

13

13

0

0

0

0

0

0

0

0

0

1

1

1

1

0

0

0

0

0

0

11

10

10

10

10

1

0

0

0

1

1

1

0

0

0

0

45

1

1

1

1

4

4

0

0

0

4

0

4

0

0

3

3

3

3

0

0

0

12

12

12

12

0

0

0

0

25

24

24

24

0

0

0

1

1

1

81

81

81

81

81

38

38

38

38

38

35

3

3

3

3

3

3

2

1

0

0

0

0

0

0

404

404

404

44

44

44

86

86

86

274

274

274

0

0

0

0

0

0

0

0

0

0

0

0

0

0

0

0

4

0

0

0

0

0

0

0

0

0

4

4

4

4

1

1

3

3

0

0

0

0

0

0

0

0

0

0

0

0
